# Physiological Influences and Hygienic Uses of Protoxide of Nitrogen

**Published:** 1864-08

**Authors:** George J. Ziegler

**Affiliations:** Philadelphia


PHYSIOLOGICAL INFLUENCES AND HYGIENIC USES OF PROTOXIDE OF NITROGEN.—*By George J. Ziegler, M. D., of Philadelphia.*—1. The *physiological influences* of nitrous oxide are of a peculiar and striking character, for though like some other agents it exerts a very energetic and decidedly stimulant action upon the animal economy, yet this is in general so entirely distinct from all other excitants as to be quite unique.

The effects of protoxide of nitrogen upon the human system vary in proportion to the quantity appropriated and the particular susceptibilities or conditions of individual organisms, passing from a gentle acceleration of all the functions of the body to a high degree of physical excitement and mental exhilaration amounting in the extreme to an intensely pleasurable delirium or ecstacy which may indeed become so pure and exquisite as to absorb the consciousness of existence itself.

When exhibited in moderate quantities nitrous oxide usually produces a mild and very pleasant thrilling sensation rapidly extending over the whole system, attended with forcible and prolonged respiratory efforts, and a strong disposition to laughter and muscular motion, which often becomes so irresistible as to be involuntary. If taken freely these agreeable sensations are greatly enhanced, accompanied with a remarkable buoyancy of spirits, boisterous gaiety, activity of imagination, rapid flow of vivid ideas and brilliancy of mental conceptions of the most sublime character, the direction of thought being influenced, in some measure, by the dominant idea at the commencement of its action, hence sometimes followed by other than hilarious manifestations. Used largely this state of excitement rapidly passes into one of rapturous enjoyment, entranced tranquility, and complete repose with entire insensibility and unconsciousness, during the existence of which the mind becomes temporarily oblivious to all impressions, even of an ordinarily painful nature. The primary stage of sur-excitation with its concomitants, anæsthesia and trance, is usually, however, of brief duration, terminating rather suddenly, yet leaving generally a sense of permanent invigoration similar to that resulting from a free exposure to fresh atmospheric air, not being followed with any reactive languor or depression so common with ordinary stimulants.

Besides its general physiological effects nitrous oxide has also special tendencies to certain parts of the body, particularly the blood, brain, nervous system, and genito-urinary organs, being very efficient in preserving the healthy integrity of such portions of the economy and in promoting the functions more immediately connected therewith.

Respecting the *modus operandi* of protoxide of nitrogen, there is very little doubt but what it exerts both a material and dynamic influence upon the animal organism through each of its constituents singly and conjointly, yet, notwithstanding the peculiar character of its biological effects seems to conclusively prove that they are dependent upon its constitutional elements in their separate as well as combined state, it is by some, thought to act through one conclusively. Nevertheless though it is obvious that much of its potency is derived from the oxygen, it is demonstrable that it alone is not sufficiently energetic to account for all of the phenomena resulting from the operation of nitrous oxide upon the human system, some of these being so entirely distinct from the usual action of that element as to justify the conclusion of the additional influence of the nitrogen for their production.

This is especially the case in its marked tendency to, and speciality of action on particular parts of the system, as for instance the genito-urinary organs, wherein it differs greatly from the former, for it only increases the excitability of these organs but improves the quality as well as regulates the quantity of secretion therefrom, both by directly augmenting their power and promoting the normal genesis and elimination of their educts. The correctness of this view is more clearly apparent from the peculiarity of the effects of nitrous oxide upon the renal secretion in the enlarged production of the nitrogenous ingredients—especially urea—of that fluid, to which it gives rise in a remarkable degree. It is most probable, therefore, that the physiological action of protoxide of nitrogen is of a compound character, from the operation of both its constituents—nitrogen and oxygen—in their associated and single state, the affinitive reactions between them and the general components of the organism being more decidedly manifested in consequence of its undergoing decomposition, and the presentation of these elements in a nascent state within the body. The chemico-organic and bio-dynamic influence of nitrous oxide is hence so much the greater from the presence of its constituents in an actively divided state, the nascent condition affording the most favorable opportunity for chemical reaction, molecular nutrition, organic metamorphosis, dynamic manifestations and vital development.

Protoxide of nitrogen is thus altogether unique in being a rapidly diffusible, potent, general, and permanent stimulant, intensifying all the vital actions, supplying elements of nutrition, exerting both a material and dynamic influence upon the animal economy, and in having a direct physiological compatibility therewith both functional and organic.

Hence, with respect to the predominant characteristics of protoxide of nitrogen, it is truly *sui generis*, though closely allied in chemical constitution, material properties and sanative effects with atmospheric air, which may be regarded, in fact, as its natural prototype, differing therefrom apparently more in the proportion of its constituent elements—nitrogen and oxygen—and in the manner of their association, than in any other essential respect, although its action upon the vital economy is more energetic, definite and perceptible than that of the latter, varying rather in the degree, perhaps, than in the nature of its physiological influence. Still, though there is thus a very strong analogy between protoxide of nitrogen and atmospheric air, yet in their relative effects upon the animal organism they vary somewhat materially, as there is a striking difference in the ratio or measure as well as in the kind of action thereupon, for the biological effects of the former are not only more intense, concentrated and manifest, but also more decidedly stimulant than those of the latter, though they are likewise of a general, very bland, and highly invigorating character.

While, therefore, there is thus a very intimate correlation between these two compound gaseous bodies—nitrous oxide and atmospheric air—yet each one has special peculiarities of its own, of such a marked character indeed as to make them appear quite distinct and render them useful for diverse as well as similar purposes.

2. The *hygienic uses* of protoxide of nitrogen are varied and important. The nature of these is indicated by the character of its physiological influences in accelerating the normal processes of life, yet for practical purposes a more specific notice thereof may not be unprofitable.

In brief, then, through its constituent elements and dynamic properties, nitrous oxide exerts a powerful influence in both supplying essential matter for organization, and in promoting the general molecular, cell, nutrient, reproductive and dynamic operations of the animal economy, those of the vegetal, animal and psychical life inclusive. It is thus, indeed, remarkably active and potent in promoting the various functions of digestion, absorption, circulation—both general and capillary—æration or arterialization, hæmatosis, calorification, assimilation, disintegration, depuration, secretion, excretion, muscular and general contractility, innervation and cerebration; and likewise those of the reproductive system. Hence for the preservation of the healthy integrity of the body and the regulation as well as invigoration of all the important functions of life, this agent may *cæteris paribus*, be always employed with advantage. This concise but comprehensive outline of the sanative effects and applications of protoxide of nitrogen will suffice to show that in an hygienic point of view it is both unique and invaluable.

It would seem, *a priori*, that an agent which exerts such a potent, material and dynamic influence over the animal organism in maintaining its normal status, should necessarily be very efficient in correcting as well as in preventing abnormal action, and both observation and experience have shown the correctness of this conclusion. It has thus been demonstrated both *a priori* and *a posteriori*, that nitrous oxide is of pre-eminent importance in restoring as well as in preserving health.

